# Association of nsv823469 copy number loss with decreased risk of chronic obstructive pulmonary disease and pulmonary function in Chinese

**DOI:** 10.1038/srep40060

**Published:** 2017-01-12

**Authors:** Xiaoliang Chen, Xiaoxiao Lu, Jiansong Chen, Di Wu, Fuman Qiu, Huali Xiong, Zihua Pan, Lei Yang, Binyao Yang, Chenli Xie, Yifeng Zhou, Dongsheng Huang, Yumin Zhou, Jiachun Lu

**Affiliations:** 1 The State Key Lab of Respiratory Disease, The Institute for Chemical Carcinogenesis, Collaborative Innovation Center for Environmental Toxicity, Guangzhou Medical University, 195 Dongfengxi Road, Guangzhou 510182, China; 2 Shenzhen Guangming district center for disease control and prevention, Shenzhen 518106, China; 3 School of Arts and Sciences, Colby-Sawyer College, New London, New Hampshire, United States of America; 4 Department of respiratory medicine, The Fifth People’s Hospital of Dongguan City, Dongguan 523900, China; 5 Department of Genetics, Medical College of Soochow University, Suzhou 215123, China

## Abstract

It is highly possible that copy number variations (CNVs) in susceptible regions have effects on chronic obstructive pulmonary disease (COPD) development, while long noncoding RNA (lncRNAs) have been shown to cause COPD. We hypothesized that the common CNV, named nsv823469 located on 6p22.1, and covering lncRNAs (major histocompatibility complex, class I, A (HLA-A) and HLA complex group 4B (HCG4B)) has an effect on COPD risk. This association was assessed through a two-stage case-control study, and was further confirmed with COPD and pulmonary function-based family analyses, respectively. The copy number loss (0-copy/1-copy) of nsv823469 significantly decreased risk of COPD compared with normal (2-copy) (OR = 0.77, 95% CI = 0.69–0.85). The loss allele, inducing copy number loss of nsv823469, has a tendency to transmit to offspring or siblings (*P* = 0.010) and is associated with forced expiratory volume in 1 second (FEV1) (*P* = 0.030). Furthermore, the copy number loss of nsv823469 in normal pulmonary tissue decreases the expression levels of HCG4B (r = 0.315, P = 0.031) and HLA-A (r = 0.296, P = 0.044). Our data demonstrates that nsv823469 plays a role in COPD and pulmonary function inheritance by potentially altering expression of HCG4B.

Multiple association studies, especially genome-wide association studies (GWASs), have revealed several susceptible loci for chronic obstructive pulmonary disease (COPD) and pulmonary function^1^,^2^,^3^. However, these loci by no means cover the complete genetic predisposition of COPD, because they mainly focus on single nucleotide polymorphism (SNP). Copy number variation (CNV)^4^,^5^ also contributes to COPD heritability^6^,^7^. CNVs can hold greater influence over covering genes than SNPs do, because CNVs can cause changes in a large DNA fragment while SNPs can only cause one base change^8^. Thus, it is highly possible that the CNVs located on susceptible regions to COPD have contributed to COPD development.

Long non-coding RNAs (lncRNAs) have been documented that play important roles in COPD^9^,^10^. Altered expression of lncRNAs, such as LINC00882, LINC00883 and PVT1, was observed in lung tissues of COPD patients^11^,^12^. A study showed that one CNV that caused disruption of LINC00299 contributed to human developmental disorders^13^. Overall, we hypothesized that the CNVs covering lncRNAs in COPD susceptible regions have effect on COPD risk.

Among the susceptible loci of COPD, only the 6p21 region was discovered by the GWAS conducted in the East Asian population^14^. The 6p21 region was included at a ~3.8 Mb interval on chromosome 6p21.32–22.1 with long-range linkage disequilibrium, and was found to have association with lung function in GWAS^15^. In reference to the published East Asian CNVs data^16^, we only found one common CNV named nsv823469 with altered copy number frequency (ACNF) > 5% located on the 6p22.1 region and covering lncRNAs. Thus, in the current study, we conducted a two-stage case-control study to find the correlation between the CNV and COPD risk, and further proved the association in two family-based analyses. The function of this CNV was further assessed by quantitative real time PCR.

## Results

### Association between the nsv823469 and risk of COPD

Significantly lower frequencies of the loss genotypes (0-copy/1-copy) were observed in cases than in controls in both the southern (*P* < 0.001) and the eastern (*P* = 0.005) Chinese population (in Table 1). According to the genetic model selection strategy based on the smallest Akaike Information Criterion (AIC) value^17^, the additive genetic model was best fitting for analysis on the effect that nsv823469 holds in COPD susceptibility. Compared to the 2-copy, the loss genotypes (0-copy/1-copy) conferred a significantly decreased risk of COPD in southern Chinese population (adjusted odds ratios (OR) = 0.77, 95% confidence interval (95% CI) = 0.68–0.87) and east Chinese population (adjusted OR = 0.76, 95% CI = 0.65–0.91). Through merging the two populations (Breslow-Day test: *P* = 0.767) in order to increase our study power, the COPD risk among the loss genotypes carriers was decreased by 23% in comparison to the 2-copy carriers (adjusted OR = 0.77, 95% CI = 0.69–0.85). Data from the stratification analysis further showed there was no significant difference among the two or three stratum-ORs (Breslow-Day test: *P* > 0.05 for all); meanwhile, no significant interaction was observed among all surrounding factors and the CNV on decreasing COPD risk (*P* > 0.05 for all; Supplementary Table S1).

### Transmission mode of nsv823469 among COPD and pulmonary function pedigrees

According to number of the mutant loss alleles, the 2-copy, 1-copy, and 0-copy genotypes were defined as wild genotypes (two normal alleles), mutant heterozygote (one normal allele and one loss allele) and mutant homozygote (two loss alleles), respectively. Family based association test (FBAT) showed that the distribution of different genotypes of nsv823469 was in accordance with that of Mendelian inheritance in families of COPD and pulmonary function^18^,^19^,^20^. The transmission disequilibrium test and sibship disequilibrium test (TDT & SDT) conducted on the 157 COPD families showed that nsv823469 had a preferential transmission of the loss allele from parents to healthy offspring or siblings under the additive genetic model (*P* = 0.010). Moreover, the loss of nsv823469 genotypes was a significant protective factor on COPD in additive genetic model (OR = 0.50, 95% CI = 0.34–0.73) as is shown in Table 2. Consistently, The quantitative transmission disequilibrium test (qTDT) conducted on the 391pulmonary function families also showed that the loss allele of nsv823469 has a tendency to transmit to offspring or siblings with relatively high forced expiratory volume in 1 second (FEV1) (*P* = 0.030; Table 3). However, no such genetic predisposition was observed for forced vital capacity (FVC) (*P* = 0.254), FEV1/FVC (*P* = 0.362) and FEV1/FEV1-predicted (*P* = 0.110).

### Effect of nsv823469 on FEV1

Basing on the transmission mode of nsv823469 introduced above, we also tested the correlation between genotypes of nsv823469 and FEV1 in all subjects of 391pulmonary function families as well as in sub-groups stratified according to categories of sex, age, smoking status, drinking status and using biomass as fuels. As shown in Table 4, the values of FEV1significantly increased along with the number of loss allele (mean ± standard deviation: 2-copy, 2.34 ± 0.81 vs. 1-copy, 2.35 ± 0.81 vs. 0-copy, 2.69 ± 0.85; K-W test: *P* = 0.001). Moreover, this trend was observed in almost all sub-groups with statistical significance except for pack-years smoked ≥ 20 packs and ever drunk due to the limited sample size.

### Effect of nsv823469 on expression of HCG4B, HLA-H, and HLA-A

**Figure 1:**
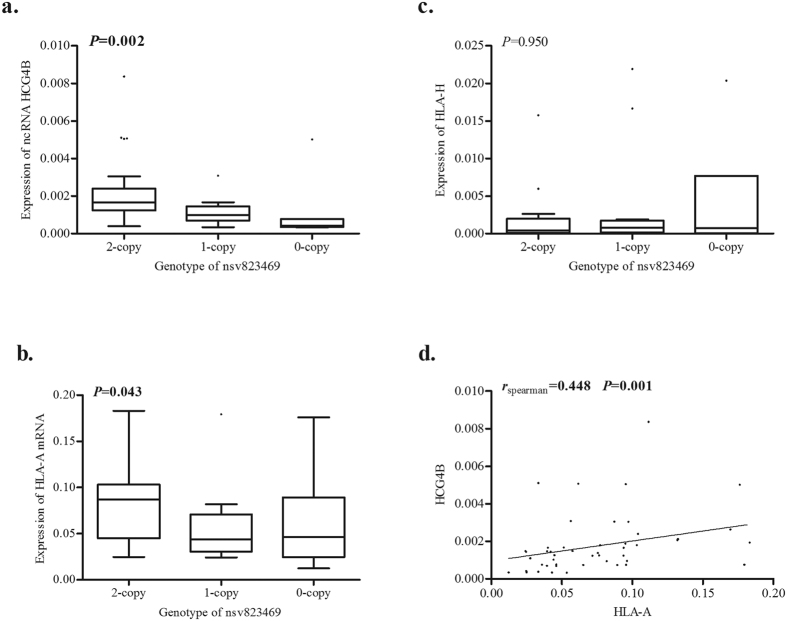
The expression of nsv823469 covering gene and lncRNA. (**a**) Effect of the CNV copy number on HCG4B; (**b**) Effect of the CNV copy number on HLA-H; (**c**) Effect of the CNV copy number on HLA-A; Bars = SD. P value was inferred with the Kruskal-Wallis test. As shown, significant deviation was observed for HCG4B and HLA-A but not HLA-H between different copy number tissues; (**d**) Correlation between the expression of HCG4B and HLA-A. A significant correlation between HCG4B and HLA-A was observed. The spearman rank correlation test was used.

Because nsv823469 covers the sequence of major histocompatibility complex, class I, H (HLA-H), major histocompatibility complex, class I, A (HLA-A), and HLA complex group 4B (HCG4B) (Supplemental Figure S1)^16^, we further tested the effect of nsv823469 on the three genes. As is shown in Fig. 1a,b, significant deviations in mRNA levels of HCG4B and HLA-A were observed in the samples of normal pulmonary tissue with different genotypes of nsv823469 (*P* = 0.002 for HCG4B and *P* = 0.043 for HLA-A). After controlled factors of sex, age and smoking by partial correlation analysis, the expression of HCG4B (*r* = 0.315, *P* = 0.031) and HLA-A (*r* = 0.296, *P* = 0.044) were still significantly positively correlated with the copy number of nsv823469. However, no significant association was observed in HLA-H with nsv823469 (*P* = 0.950, Fig. 1c). It means that copy number loss of nsv823469 significantly decreased the expression of HLA-A and HCG4B. Furthermore, we found that the expression of HCG4B was significantly correlated with that of HLA-A (*r* = 0.448, *P* = 0.001; Fig. 1d), while HCG4B was not significantly correlated with major histocompatibility complex, class I, F (HLA-F), major histocompatibility complex, class I, G (HLA-G), and major histocompatibility complex, class I, J (HLA-J) (*P* > 0.05 for all; Supplementary Figure S2a–c). In addition, the CNV has no effect on HLA-F, HLA-G and HLA-J expressions, as is expected (*P* > 0.05 for all; Supplementary Figure S2d–f).

### Prediction of mechanism on HCG4B mediating HLA-A

Because some similarities were found between the mRNA sequence of HCG4B and that of HLA-A, bioinformatics analysis was performed to deduce a possible molecular mechanism through the website miRcode (http://mircode.org/index.php), which identifies putative target sites base on seed complementarity and evolutionary conservation^21^. As is shown in Supplementary Table S2, HCG4B might act as a competing endogenous RNAs (ceRNA) spongingmiR-122 and miR-1352 to increase the HLA-A expression.

## Discussion

Based on a two-stage case-control study and two family based analyses, nsv823469 was identified to be associated with decreased risk of COPD in Chinese, and the loss allele has a tendency to transmit to health offspring/sibling and those with relatively high FEV1. Functional analysis further showed the CNV has effect on HLA-A and lncRNA HCG4B expression, meanwhile HCG4B could regulate the expression of HLA-A.

Now the function of HCG4B remains unknown. In the current study, we found that the expression of HCG4B was positively correlated with that of HLA-A, which suggested that HCG4B may regulate the expression of HLA-A. The molecular mechanism may be that HCG4B acts as a competing endogenous RNAs (ceRNA) sponging miR-122 and miR-1352. HLA-A plays an important role in COPD development with respect to immune function, and has been identified to have high expression in alveolar epithelial type II cells (ATII cells) and higher frequency in peripheral blood lymphocytesin COPD patients to mediate the development of COPD^22^,^23^,^24^. Moreover, evidences have supported that HLA-A participates in CD8 T cell involving in apoptosis of lung cells in the pathological process of COPD^24^. Based on the foregoing evidence, it is functionally possible that the loss copies of nsv823469 conferred a decreased risk of COPD.

Consistently, both the case-control study and COPD family-based analysis demonstrated significantly decreased risk in subjects with loss copies in comparison to those with 2-copy. Furthermore, nsv823469 had a preferential transmission of loss allele towards health children or siblings from parents. Moreover, the pulmonary function-based family analysis showed that the loss allele has a tendency to transmit to offspring/siblings with relatively high FEV1. Altogether, nsv823469 contributes to COPD predisposition and phenotypic pleiotropy. In addition, the family-based designs gave high credibility to our findings, because these designs could effectively control the confounding effects caused by various confounders in the case-control studies widely seen in association studies^25^.

As of now, only three studies have examined the associations between genomic CNVs and COPD risk^4^,^26^,^27^, and all of them focused on coding genes. In our study, we paid close attention to lncRNA CNVs that are located in the susceptible regions of COPD. We further found this CNV’s association with COPD risk in Chinese through the mechanism of regulating the expression of the lncRNA HCG4B and followed HLA-A. To our best knowledge, this is the first study investigating CNVs crossing with lncRNA on COPD risk.

There are also some limitations to the current study. Firstly, based on case-control study and family-based analyses, biases, such as selection bias and information bias, cannot be completely ruled out. Secondly, limited by the capabilities of the various technologies, we did not reveal the molecular function of the lncRNA on COPD development. We also did not substantiate the exact mechanism of how HCG4B influences the HLA-A expression. Nevertheless, all studies here exerted consistent results that the CNV has functional association with COPD risk and lung function, and it strongly suggests such association is not achieved by chance.

In summary, our study identified a putatively functional CNVnsv823469 that conferred declined risk of COPD and was beneficial to pulmonary function. The CNV underlies a biological mechanism that it could induce a low expression of HCG4B and followed HLA-A. Taken together, the CNV nsv823469 might be a genetic biomarker to predict risk of COPD in Chinese.

## Methods

### Case-control study

As described in previously published studies^5^,^28^,^29^, a two-stage case-control study was conducted in the southern Chinese and eastern Chinese population. In brief, 1025 COPD patients and 1061 controls were enrolled from Guangzhou city; 486 COPD patients and 616 controls were recruited from Suzhou city. COPD was diagnosed according to the Global initiative for chronic Obstructive Lung Disease (GOLD) criterion of FEV1/FVC < 70% after inhalation of 400 μg salbutamol^30^. The controls with FEV1/FVC > 70% were age (±5 years) and sex frequency-matched with the cases. The subjects donated 5-mL peripheral blood after giving their informed consents, and were interviewed using a structured questionnaire to provide data on demographic variables and risk factors. Their frequency distributions in case and control groups have been described in our previous publication^28^ (Supplementary Table S3). This study was approved by the institutional review boards of Guangzhou Medical University and Soochow University.

### COPD family based analysis

A COPD family based analysis was conducted in the southern Han Chinese population between September 2010 and March 2015. 157 COPD probands were firstly enrolled and their immediate family members, including parents, siblings, and offspring, were asked to take a COPD diagnostic test. Excluding those who did not finish the lung function test, 293 immediate family members were ultimately enrolled, among which 44 were diagnosed with COPD while 249 were healthy. All subjects were interviewed using the above questionnaire, and donated 5-ml peripheral blood after signing the informed consent. This study was approved by the institutional review boards of Guangzhou Medical University.

### Family based pulmonary function analysis

A family based pulmonary function analysis on community individuals was conducted between May 2014 and May 2015 in the southern Han Chinese. By excluding those who had no immediate family members or whose immediate family members did not complete lung function test, 391 pulmonary function relative families (n = 987) were finally recruited from annual cross-sectional surveys of COPD. Each subject donated 5 ml peripheral blood and was interviewed to provide data on the above variables after writing an informed consent. The study was approved by the institutional review boards of Guangzhou Medical University.

### CNV selection and genotyping

By referring to East Asian CNVs data^16^, 15 CNVs were found in the region 6p21.32–22.1. Among them, only one CNV named CNVR2829.8 was prevalent in East Asian with the ACNF > 5%. The CNV was also recorded as nsv823469 in the database of genomic variants (DGV: http://dgv.tcag.ca/dgv/app/home). For the purpose of unequivocal reference, we used the label nsv823469 throughout the current study. The genotype of nsv823469 was detected by the TaqMan assay with special probes and primers (FAM labeled, cat no. Hs03587795; proprietary technology of Applied Biosystems) from the ABI by life Technology Company^31^ according to the standard protocol. The genotype was automatically determined by software Copy Caller 2.1 (Applied Biosystems; Supplementary Figure S3).

### Detection of nsv823469 covering genes’ mRNA levels

According to the DGV database and East Asian CNVs data^16^, the loss of nsv823469 causes lower copies or deletion of DNA sequence that covers two lncRNAs with names of HLA-H and HCG4B, and some sequence of a coding-gene HLA-A in East Asian. The loss of nsv823469 may decrease the expressions of HCG4B, HLA-H and HLA-A with the dosage effect. Thus, we tested the mRNA levels of the above genes and a reference gene *β-actin* in 50 samples of normal pulmonary tissue using SYBR-Green real-time PCR, as the samples were obtained from the tumor hospital affiliated to Guangzhou Medical University with the characteristics shown in Supplementary Table S4. The expressions of HLA-F, HLA-G and HLA-J were also detected because of their genomic locations approaching to the HCG4B, while these genes might be the target genes of HCG4B. The primers for each gene presented in the Supplementary Table S5. Each sample was run in triplicate and the mean level of mRNA was calculated. Moreover, the genotype of nsv823469 for each sample was detected by the TaqMan assay.

### Informed consent for Using experimental animals and human subjects

This study obtained the consents of all participants and fit in the standard moral principles of human beings and all experiments were performed in accordance with relevant guidelines and regulations. Furthermore, this study was approved by the institutional review boards of Guangzhou Medical University (Ethics Committee of Guangzhou Medical University: GZMC2007–07–0676) and Soochow University (Ethics Committee of Soochow University: SZUM2008031233).

### Statistical analysis

The *χ*^2^ test was used to evaluate the consistency of loss frequency of nsv823469 in East Asian samples (11/30)^16^ and controls of the case-control study. The unconditional logistic regression model was applied in order to estimate the association strength between the CNV and COPD risk. The homogeneity of genetic effects among each stratum was analyzed with Breslow-Day test. Interactions between the nsv823469 and surrounding factors were assessed using the multiplicative interaction analysis. TDT & SDT of the nsv823469 among COPD families were analyzed using the FBAT software. qTDT of nsv823469 among pulmonary function families was performed on FEV1, FVC, FEV1/FVC and FEV1/ FEV1-predicted^32^ by the FAMILY PROCEDURE in SAS(http://support.sas.com/documentation/cdl/en/geneug/64249/HTML/default/viewer.htm#geneug_family_sect023.htm). Furthermore, the Kruskal-Wallis test and Spearman rank correlation test were performed to assess the effect nsv823469 has on pre-bronchodilator lung function traits and mRNA expressions of HCG4B, HLA-H and HLA-A. All tests were two-sided ones, and *P* < 0.05 was considered to be statistically significant.

## Additional Information

**How to cite this article**: Chen, X. *et al*. Association of nsv823469 copy number loss with decreased risk of chronic obstructive pulmonary disease and pulmonary function in Chinese. *Sci. Rep.* **7**, 40060; doi: 10.1038/srep40060 (2017).

**Publisher's note:** Springer Nature remains neutral with regard to jurisdictional claims in published maps and institutional affiliations.

## Supplementary Material

Supplementary Information

## Figures and Tables

**Table 1:** Association between the nsv823469 copy numbers and COPD risk in case-control studies.

Genotype/Allele	Case	Control	*P*^a^	Crude	Adjusted
	n (%)	n (%)		OR (95% CI)	OR (95% CI)^b^
Southern Chinese	1025	1061	<0.001		
Co-dominant model^c^					2895.2
2-copy	690 (67.32)	642 (60.51)		1.00 (ref.)	1.00 (ref.)
1-copy	232 (22.63)	244 (23.00)		0.87 (0.71–1.08)	0.88 (0.71–1.09)
0-copy	103 (10.05)	175 (16.49)		0.54 (0.42–0.71)	0.55 (0.42–0.72)
Additive model^c^					2876.2
0 vs. 1 vs. 2				0.77 (0.68–0.87)	0.77 (0.68–0.87)
Dominant model^c^					2884.7
(0 + 1) vs. 2				0.74 (0.62–0.89)	0.74 (0.62–0.89)
Recessive model^c^					2877.4
(1 + 2) vs. 0				1.77 (1.36–2.29)	1.76 (1.35–2.29)
Eastern Chinese	486	616	0.005		
Co-dominant model^c^					1516.3
2-copy	333 (68.52)	374 (60.71)		1.00 (ref.)	1.00 (ref.)
1-copy	111 (22.84)	153 (24.84)		0.82 (0.61–1.08)	0.89 (0.66–1.21)
0-copy	42 (8.64)	89 (14.45)		0.53 (0.36–0.79)	0.53 (0.37–0.77)
Additive model^c^					1504.4
0 vs. 1 vs. 2				0.75 (0.63–0.90)	0.76 (0.65–0.91)
Dominant model^c^					1509.1
(0 + 1) vs. 2				0.71 (0.55–0.91)	0.73 (0.57–0.94)
Recessive model^c^					1504.7
(1 + 2) vs. 0				1.85 (1.30–2.65)	1.81 (1.26–2.60)
Merged above population	1511	1677	<0.001		
Co-dominant model^c^					4414.8
2-copy	1023 (67.70)	1016 (60.58)		1.00 (ref.)	1.00 (ref.)
1-copy	343 (22.70)	397 (23.67)		0.88 (0.74–1.04)	0.88 (0.74–1.05)
0-copy	145 (9.60)	264 (15.75)		0.54 (0.43–0.67)	0.54 (0.44–0.68)
Additive model^c^					4383.7
0 vs. 1 vs. 2				0.76 (0.69–0.84)	0.77 (0.69–0.85)
Dominant model^c^					4397.3
(0 + 1) vs. 2				0.73 (0.63–0.85)	0.74 (0.64–0.86)
Recessive model^c^					4385.3
(1 + 2) vs. 0				1.80 (1.46–2.22)	1.79 (1.44–2.21)

^a^*P* values of a two-sided χ^2^ test for genotypes distribution between the cases and controls.

^b^Adjusted in a logistic regression model with age, sex, smoking, biomass as fuels and drinking.

^c^Akaike information criterion (AIC) value.

**Table 2:** FBAT analysis of the nsv823469 copy numbers in 157 COPD pedigrees families.

Allele	Afreq	Fam#	S-E(S>)	Var(S)	Z	*P*	Logistic regression model^a^	
							P	OR (95% CI)
Loss allele	0.232	48	9.917	14.862	2.572	0.010102	<0.001	0.50 (0.34–0.73)
Normal allele	0.768	48	−9.917	14.862	−2.572	0.010102		

Abbreviation: fam#: Number of nuclear families informative for the FBAT analysis (at least one parent must be heterozygous). S: Observed transmission for each allele; E(S) = Expected transmission for each allele. Var(S): Variance of the observed transmission for each allele.

^a^Adjusted by age, sex, smoking, biomass as fuels and drinking under additive model.

**Table 3:** The pulmonary function traits on nsv823469 and heritability of it in 391 families.

Traits	The distribution of lung function traits on nsv823469							Family based analysis	
	0-copy		1-copy		2-copy		Kruskal-Wallis test	qTDT test^a^	
	N	M ± SE	N	M ± SE	N	M ± SE	*P*	Z	*P*
FEV1	110	2.69 ± 0.85	279	2.35 ± 0.81	598	2.34 ± 0.81	0.001	**2.17**	**0.030**
FVC	110	3.24 ± 0.94	279	2.93 ± 0.9	598	3.01 ± 0.93	0.012	1.91	0.056
FEV1/FVC	110	0.83 ± 0.07	279	0.79 ± 0.09	598	0.77 ± 0.12	<0.001	1.20	0.056
FEV1/FEV1-predicted	110	0.85 ± 0.22	279	0.79 ± 0.16	598	0.78 ± 0.17	<0.001	1.79	0.073

Abbrev: Mean ± Standard Error, M ± SE.

^a^Calculated by Rabinowitz formulas.

**Table 4:** Effect of the CNVnsv823469 on FEV1 in total 987 subjects from the 391 families.

Variants	2-copy		1-copy		0-copy		Kruskal-Wallis test
	n	M ± SE	n	M ± SE	n	M ± SE	*P* value
Total	597	2.34 ± 0.81	280	2.35 ± 0.81	110	2.69 ± 0.85	0.001
Sex							
Male	275	2.69 ± 0.90	126	2.77 ± 0.86	52	3.15 ± 0.83	0.003
Female	322	2.05 ± 0.63	154	2.01 ± 0.56	58	2.28 ± 0.64	0.015
Age							
≤60years	426	2.64 ± 0.71	197	2.64 ± 0.73	82	2.91 ± 0.78	0.023
>60years	171	1.61 ± 0.60	83	1.65 ± 0.52	28	2.04 ± 0.75	0.010
Smoking status							
Ever smoked	145	2.45 ± 0.90	67	2.61 ± 0.88	24	3.16 ± 0.83	0.003
Never smoked	452	2.31 ± 0.8	213	2.26 ± 0.77	86	2.56 ± 0.82	0.020
Pack-years smoked(packs)							
≥20	55	2.25 ± 0.83	27	2.23 ± 0.77	9	2.63 ± 0.87	0.528
0<packs<20	90	2.58 ± 0.92	40	2.86 ± 0.87	15	3.47 ± 0.63	0.002
Using biomass as fuels							
Ever used	410	2.24 ± 0.74	199	2.27 ± 0.78	80	2.52 ± 0.77	0.021
Never used	187	2.59 ± 0.95	81	2.54 ± 0.86	30	3.13 ± 0.92	0.015
Drinking status							
Ever drunk	115	2.78 ± 0.75	57	2.74 ± 0.82	31	2.87 ± 0.92	0.835
Never drunk	482	2.24 ± 0.81	223	2.24 ± 0.78	79	2.62 ± 0.82	0.001

Abbrev: Mean ± Standard Error, M ± SE.
