# Supplementary material for: Association of nsv823469 copy number loss with decreased risk of chronic obstructive pulmonary disease and pulmonary function in Chinese
Source: Sci Rep. 2017 Jan 12;7:40060. doi: 10.1038/srep40060 (PMC5227687; doi:10.1038/srep40060)
Supplement: Supplementary Information [file srep40060-s1.pdf]

**Association of nsv823469 copy number loss with decreased risk of chronic obstructive pulmonary disease and pulmonary function in Chinese**

Xiaoliang Chen[1,2], Xiaoxiao Lu[3], Jiansong Chen[1], Di Wu[1], Fuman Qiu[1], Huali Xiong[1], Zihua Pan[1], Lei Yang[1], Binyao Yang[1], Chenli Xie[4], Yifeng Zhou[5], Dongsheng Huang[1], Yumin Zhou[1], Jiachun Lu[1§]

[1]The State Key Lab of Respiratory Disease, The Institute for Chemical Carcinogenesis, Collaborative Innovation Center for Environmental Toxicity, Guangzhou Medical University, 195 Dongfengxi Road, Guangzhou 510182, China.

[2] Shenzhen Guangming district center for disease control and prevention Shenzhen 518106, China.

[3]School of Arts and Sciences, Colby-Sawyer College, New London, New Hampshire, United States of America.

[4]Department of respiratory medicine, The Fifth People's Hospital of Dongguan City, Dongguan 523900, China.

[5]Department of Genetics, Medical College of Soochow University, Suzhou 215123, China.

**§Corresponding author:**

Dr. Jiachun Lu, The State Key Lab of Respiratory Disease, The Institute for Chemical Carcinogenesis, Collaborative Innovation Center for Environmental Toxicity, Guangzhou Medical University, 195 Dongfengxi Road, Guangzhou 510182, P.R. China. Tel: 86-20-37104661; Fax: 86-20-37104661; E-mail: jcLu@gzhmu.edu.cn

Supplemental Figures:

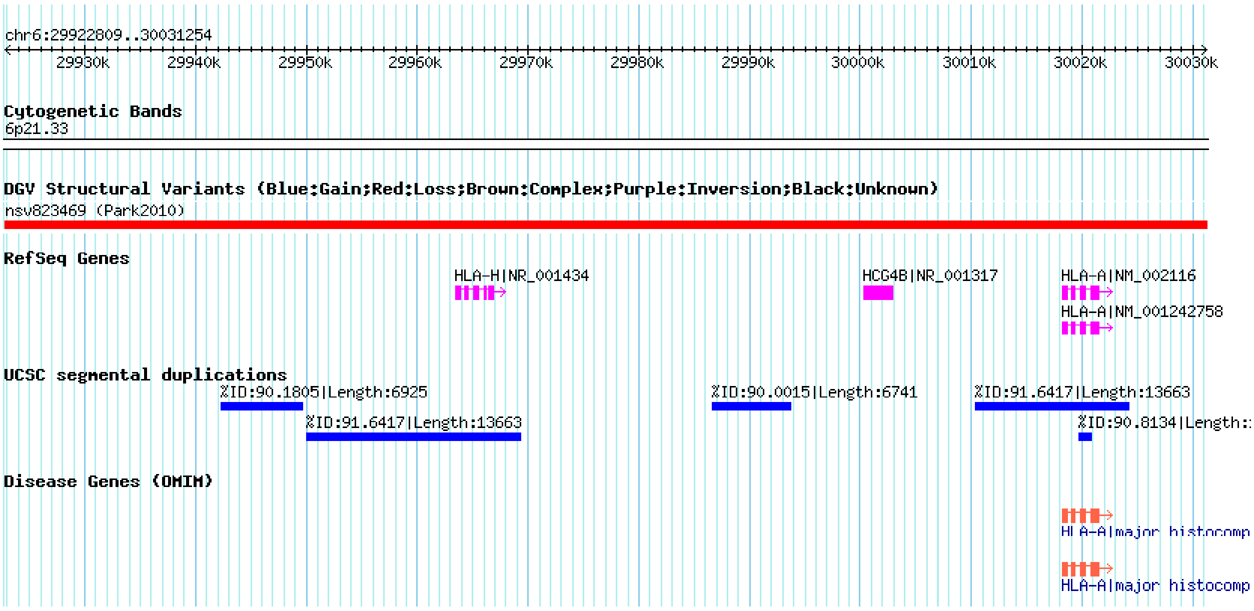


**Supplementary Figure S1.** The location of nsv823469 in chromosome and included genes.

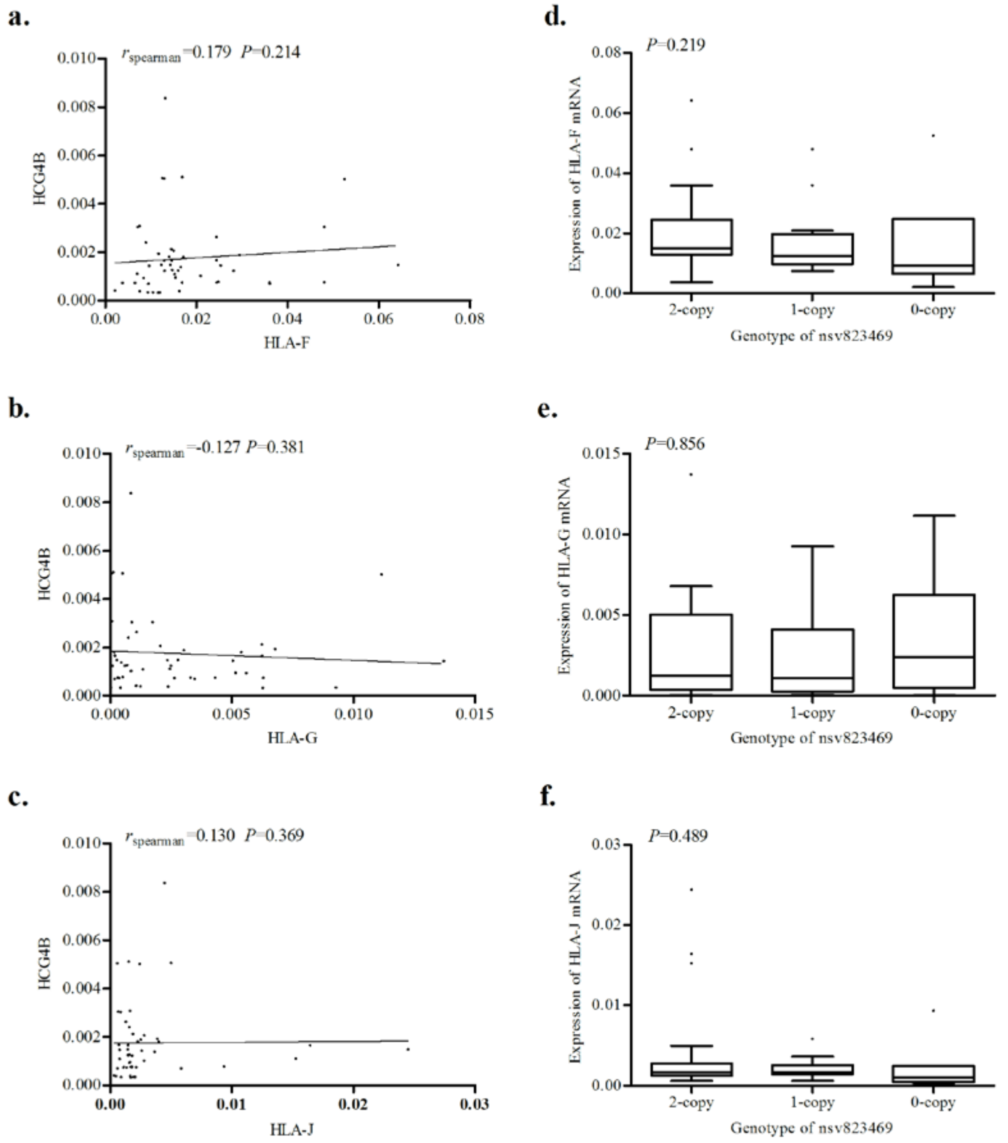


**Supplementary Figure S2.** The expressions of HLA-F, HLA-G and HLA-J in corresponding non-tumor normal tissues. a. Correlation between the expression of HCG4B and HLA-F; b. Correlation between the expression of HCG4B and HLA-G; c. Correlation between the expression of HCG4B and HLA-J. No significant correlation was observed between HCG4B and HLA-F, HLA-G and HLA-J. The spearman rank correlation test was used. d. Effect of the nsv823469 copy number on HLA-F expression; e. Effect of the nsv823469 copy number on HLA-G expression; f. Effect of the nsv823469 copy number on HLA-J expression. Bars = SD. P value was inferred with the Kruskal-Wallis test.

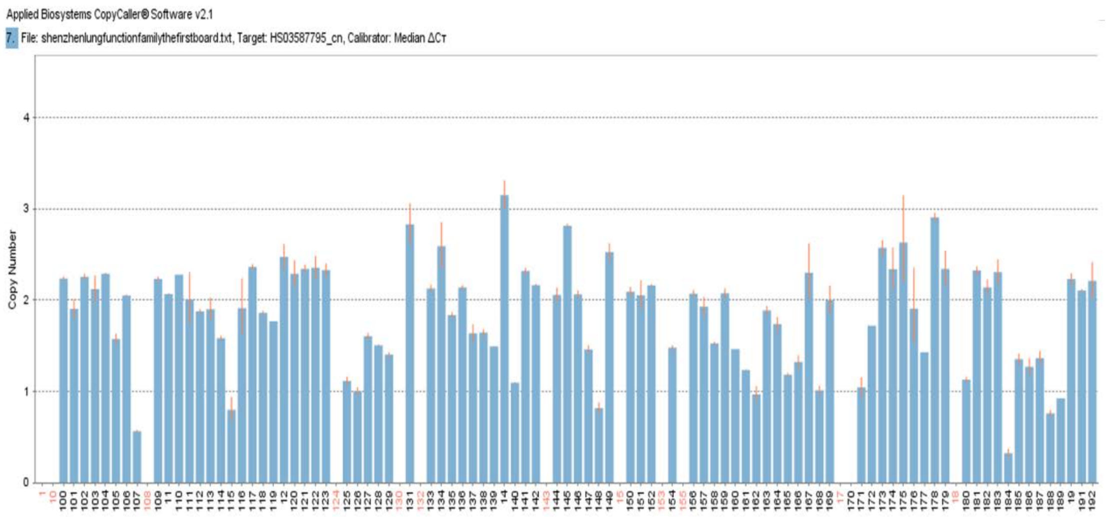


**Supplementary Figure S3.** The genotyping of CNV nsv823469 by the TaqMan assay. The copy number was automatically determined by the software Copy Caller 2.1 in ABI 7900HT

**Supplementary Table S1.** Stratification analysis of the association between nsv823469 and COPD risk.

| Factors | Case ($n$ = 1511) | | | Control ($n$ = 1677) | | | Adjusted OR ( 95% CI) [a] (0-copy+1-copy) vs. 2-copy | $P^b$ | $P^c$ |
|---|---|---|---|---|---|---|---|---|---|
| | 0-copy | 1-copy | 2-copy | 0-copy | 1-copy | 2-copy | | | |
| Age (Years) | | | | | | | | | |
| ≤ 60 | 74 | 153 | 477 | 138 | 176 | 485 | 0.75(0.60-0.93) | 0.97 | 0.49 |
| > 60 | 79 | 182 | 546 | 145 | 202 | 531 | 0.74(0.60-0.91) | | |
| Sex | | | | | | | | | |
| Male | 84 | 198 | 601 | 174 | 221 | 588 | 0.69(0.57-0.85) | 0.44 | 0.38 |
| Female | 69 | 137 | 422 | 109 | 157 | 428 | 0.81(0.64-1.02) | | |
| Smoking status | | | | | | | | | |
| Never | 82 | 179 | 536 | 159 | 233 | 634 | 0.80(0.66-0.98) | 0.51 | 0.37 |
| Ever | 71 | 156 | 487 | 124 | 145 | 382 | 0.67(0.54-0.84) | | |
| Pack-years smoked | | | | | | | | | |
| ≥20 | 44 | 119 | 334 | 85 | 82 | 216 | 0.64(0.49-0.86) | 0.41 | 0.08 |
| <20 | 27 | 37 | 153 | 39 | 63 | 166 | 0.71(0.48-1.05) | | |
| 0 | 82 | 179 | 536 | 159 | 233 | 634 | 0.80(0.66-0.98) | | |
| Biomass as fuels | | | | | | | | | |
| No | 138 | 286 | 880 | 268 | 349 | 950 | 0.74(0.63-0.87) | 0.70 | 0.72 |
| Yes | 15 | 49 | 143 | 15 | 29 | 66 | 0.68(0.41-1.11) | | |
| Drinking status | | | | | | | | | |
| Never | 120 | 275 | 833 | 219 | 300 | 822 | 0.75(0.64-0.89) | 0.75 | 0.55 |
| Ever | 33 | 60 | 190 | 64 | 78 | 194 | 0.67(0.47-0.93) | | |
| Gold Stages | | | | | | | | | |
| I | 77 | 130 | 414 | 283 | 378 | 1016 | 0.75(0.64-0.89) | 0.77 | |
| II | 51 | 131 | 412 | | | | 0.68(0.49-0.96) | | |
| III | 18 | 51 | 142 | | | | 0.64(0.48-0.85) | | |
| IV | 7 | 23 | 55 | | | | 0.67(0.54-0.85) | | |

[a]ORs were adjusted for age, sex, smoking status and biomass as fuels and drinking status in a logistic regression models.
[b]P value of Breslow-Day Test.
[c]*P* value of test for the multiplicative interaction between CNV of HCG4B and selected variables on COPD risk in logistic regression model.

**Supplementary table S2.** The predicted miRNAs on targeted genes of primates in miRcode

| genes | microRNA family | Seed position | Seed type | Transcript region |
|---|---|---|---|---|
| HCG4B | let-7/98/4458/4500 | chr6:29894347 | 7-mer-m8 | ncRNA |
| | **miR-122/122a/1352**[#] | chr6:29892660 | 8-mer | ncRNA |
| | miR-132/212/212-3p | chr6:29892722 | 7-mer-m8 | ncRNA |
| | miR-135ab/135a-5p | chr6:29894978 | 7-mer-A1 | ncRNA |
| | miR-139-5p | chr6:29892940 | 7-mer-A1 | ncRNA |
| | miR-145 | chr6:29893803 | 7-mer-m8 | ncRNA |
| | miR-150/5127 | chr6:29894068 | 8-mer | ncRNA |
| | miR-155 | chr6:29893438 | 7-mer-m8 | ncRNA |
| | miR-181abcd/4262 | chr6:29892540 | 8-mer | ncRNA |
| | miR-193/193b/193a-3p | chr6:29893263 | 7-mer-A1 | ncRNA |
| | miR-1ab/206/613 | chr6:29893351 | 7-mer-A1 | ncRNA |
| | miR-200bc/429/548a | chr6:29893071 | 7-mer-A1 | ncRNA |
| | miR-217 | chr6:29893221 | 7-mer-m8 | ncRNA |
| | miR-33ab/33-5p | chr6:29893223 | 7-mer-m8 | ncRNA |
| | miR-425/425-5p/489 | chr6:29892680 | 7-mer-m8 | ncRNA |
| | miR-503 | chr6:29894385 | 7-mer-m8 | ncRNA |
| | miR-551a | chr6:29894830 | 7-mer-A1 | ncRNA |
| | miR-96/507/1271 | chr6:29893928 | 7-mer-m8 | ncRNA |
| HLA-A | miR-10abc/10a-5p | chr6:29910184 | 7-mer-m8 | 5pUTR |
| | **miR-122/122a/1352**[#] | chr6:29910545 | 7-mer-A1 | CDS,5pUTR,ncRNA |
| | miR-124/124ab/506 | chr6:29912103 | 7-mer-m8 | 3pUTR,CDS,ncRNA |
| | miR-146ac/146b-5p | chr6:29910222 | 8-mer | 5pUTR |
| | miR-146ac/146b-5p | chr6:29911051 | 7-mer-A1 | CDS,5pUTR,ncRNA |
| | miR-148ab-3p/152 | chr6:29913504 | 8-mer | 3pUTR,ncRNA |
| | miR-184 | chr6:29912497 | 7-mer-A1 | ncRNA |
| | miR-187 | chr6:29910727 | 7-mer-m8 | CDS,5pUTR,ncRNA |
| | miR-199ab-5p | chr6:29912353 | 7-mer-A1 | 3pUTR,CDS,ncRNA |
| | miR-205/205ab | chr6:29909060 | 7-mer-A1 | 5pUTR |
| | miR-205/205ab | chr6:29912573 | 8-mer | ncRNA |
| | miR-205/205ab | chr6:29912836 | 7-mer-A1 | CDS,ncRNA |
| | miR-216a | chr6:29912427 | 7-mer-m8 | ncRNA |
| | miR-219-5p/508/508-3p/4782-3p | chr6:29912503 | 7-mer-A1 | ncRNA |
| | miR-23abc/23b-3p | chr6:29910735 | 8-mer | CDS,5pUTR,ncRNA |
| | miR-23abc/23b-3p | chr6:29913361 | 7-mer-m8 | 3pUTR,ncRNA |
| | miR-26ab/1297/4465 | chr6:29913296 | 7-mer-A1 | 3pUTR,ncRNA |
| | miR-31 | chr6:29913573 | 8-mer | 3pUTR,ncRNA |
| | miR-490-3p | chr6:29911048 | 7-mer-m8 | ncRNA |
| | miR-7/7ab | chr6:29912288 | 7-mer-m8 | 3pUTR,CDS,ncRNA |
| | miR-93/93a/105/106a/291a-3p/294/295/ 302abcde/372/373/428/519a/520be/ 520acd-3p/1378/1420ac | chr6:29912541 | 8-mer | ncRNA |

[#] the same miRNAs for HCG4B and HLA-A.

**Supplementary Table S3.**Frequency distributions of demographic characteristics, possible risk factors and GOLD stages in COPD cases and controls.

| Variables | Southern Chinese | | | Eastern Chinese | | |
|---|---|---|---|---|---|---|
| | Cases n (%) | Controls n (%) | *P* | Cases n (%) | Controls n (%) | *P* |
| Total No. of subjects | 1025 | 1061 | | 486 | 616 | |
| Age (years) | | | | | | |
| ≤ 60 | 463(45.2) | 507(47.8) | 0.231 | 241(49.6) | 292(47.4) | 0.471 |
| > 60 | 562(54.8) | 554(52.2) | | 245(50.4) | 324(52.6) | |
| Sex | | | | | | |
| Male | 610(59.5) | 638(60.1) | 0.773 | 273(56.2) | 345(56.0) | 0.956 |
| Female | 415(40.5) | 423(39.9) | | 213(43.8) | 271(44.0) | |
| Smoking status | | | | | | |
| Ever | 499(48.7) | 424(40.0) | <0.001 | 215(44.2) | 227(36.8) | 0.013 |
| Never | 526(51.3) | 637(60.0) | | 271(55.8) | 389(63.2) | |
| Pack-years smoked | | | | | | |
| ≥20 | 314(30.6) | 214(20.2) | <0.001 | 148(30.5) | 129(20.9) | 0.025 |
| <20 | 185(18.1) | 210(19.8) | | 67(15.2) | 98(15.9) | |
| 0 | 526(51.3) | 637(60.0) | | 271(55.8) | 389(63.2) | |
| Using biomass as fuels | | | | | | |
| Yes | 174(17.0) | 91(8.6) | <0.001 | 33(6.8) | 19(3.1) | 0.004 |
| No | 851(83.0) | 970(91.4) | | 453(93.2) | 597(96.9) | |
| Drinking status | | | | | | |
| Ever | 186(18.2) | 209(19.7) | 0.366 | 97(20.0) | 127(20.6) | 0.788 |
| Never | 839(81.8) | 852(80.3) | | 389(80.0) | 489(79.4) | |
| Gold stages | | | | | | |
| I | 359(35.0) | | | 213(43.8) | | |
| II | 356(34.7) | | | 206(43.4) | | |
| III | 217(21.2) | | | 54(11.1) | | |
| IV | 93(9.1) | | | 13(2.7) | | |

**Supplementary Table S4.** The charecteristics of 50 corresponding non-tumor normal tissues

| Variables | n | % |
|---|---|---|
| Total NO. of subjects | 50 | 100 |
| Sex | | |
| Male | 36 | 72 |
| Female | 14 | 28 |
| Age (years) | | |
| ≤60 | 30 | 60 |
| >60 | 20 | 40 |
| Smoking status | | |
| Ever | 24 | 48 |
| Never | 26 | 52 |
| Type of diagnosis | | |
| Pulmonary adenocacinoma | 15 | 30 |
| Pulmonary squamous cell carcinoma | 28 | 56 |
| Pulmonary benign tumor | 6 | 12 |
| Pulmonary hamartoma | 1 | 2 |

**Supplementary Table S5.**The primers of RT-PCR for detecting the expressions of the target genes and LncRNA in the normal pulmonary tissue.

| gene | name of primer | Sequence(5'to3') | length of sequence |
|---|---|---|---|
| HCG4B | HCG4B-F | CAGCCCTGAGTTACTGGGTG | 20 |
| HCG4B | HCG4B-R | GAGCCTCGCTCAGTGTTCTT | 20 |
| HLA-H | HLA-H-F | TATTGGGACCGGAACACACA | 20 |
| HLA-H | HLA-H-R | CGTAGGCGTGCTGTTCATAC | 20 |
| HLA-F | HLA-F-F | GGAATGAATGGCTGCGACAT | 20 |
| HLA-F | HLA-F-R | TCCTCTGCCTCATAGAAGCG | 20 |
| HLA-G | HLA-G-F | TTGCAGCTGTAGTCACTGGA | 20 |
| HLA-G | HLA-G-R | AGAGGAGGAATTGTGGGGTG | 20 |
| HLA-A | HLA-A-F | GCAAGGATTACATCGCCCTG | 20 |
| HLA-A | HLA-A-R | ATGGGGTGGTGGGTCATATG | 20 |
| HLA-J | HLA-J-F | CTACCCTGCGGAGATCACAT | 20 |
| HLA-J | HLA-J-R | GGACCCTCGAGAAGTTTCCA | 20 |
| β-actin | β-actin-F | GGCGGCACCACCATGTACCCT | 21 |
| β-actin | β-actin-R | AGGGGCCGGACTCGTCATACT | 21 |
